# Editorial for the Special Issue on the Latest Advancements in Semiconductor Materials, Devices and Systems

**DOI:** 10.3390/mi15121422

**Published:** 2024-11-27

**Authors:** Xinghuan Chen, Fangzhou Wang, Zirui Wang, Zeheng Wang, Jing-Kai Huang

**Affiliations:** 1 The China Electronic Product Reliability and Environmental Testing Research Institute, Guangzhou 510610, China; 2 Songshan Lake Materials Laboratory, Dongguan 523808, China; 3 School of Integrated Circuits, Peking University, Beijing 100871, China; 4 Manufacturing, CSIRO, Lindfield, NSW 2070, Australia; 5 Department of Systems Engineering, The City University of Hong Kong, Kowloon, Hong Kong 999077, China; 6 School of Materials Science and Engineering, University of New South Wales (UNSW), Sydney, NSW 2052, Australia

The field of semiconductor research is experiencing a paradigm shift as the boundaries of Moore’s Law are being approached [1]. While the exploration of next-generation semiconductors, such as 2D materials and wide-bandgap semiconductors, continues to reveal new horizons for electronic devices, advancements in traditional semiconductors, such as silicon (Si) and germanium (Ge), remain crucial for current and emerging technologies [2,3,4,5]. These advancements, in conjunction with sophisticated modeling, simulation, and fabrication techniques, are paving the way for remarkable innovations not only in materials and devices but also in circuits and systems, thus enriching the landscape of semiconductor research. In this Editorial, we summarize 10 cutting-edge papers that highlight the wide range of innovations within the design, optimization, and modeling of devices and systems based on various materials, as well as their applications. These contributions can be broadly categorized into five key areas: (1) revolutionary materials, (2) the optimization of devices, (3) advanced modeling methods, (4) the techniques used for characterizing traps, and (5) circuits and systems.

The creation of wide- and ultrawide-bandgap semiconductor materials, such as silicon carbide (SiC), gallium nitride (GaN), and diamond, has significantly improved the performance of power electronics [6,7,8]. Rafin et al. provided a comprehensive review of wide- and ultrawide-bandgap semiconductor power devices, comparing Si, SiC, GaN, and emerging diamond technology devices [9]. Wide- and ultrawide-bandgap semiconductor power devices exhibit significant superiority over Si in terms of their voltage blocking, switching speeds, efficiency, and thermal performance. SiC and GaN devices are becoming increasingly prevalent, particularly in electric vehicles, renewable energy, aerospace, and high-frequency applications. Diamond’s exceptionally wide bandgap could enable unprecedented power densities and the high-temperature operation of power devices.

GaN-based high-electron-mobility transistors (HEMTs) are considered promising candidates for next-generation high-efficiency power conversion applications [10,11,12,13]. The optimization of these devices is critical to improving their performance [14]. Chen et al. present a novel enhancement-type GaN HEMT with a high power transmission capability [15]. In this transistor, a graded Al mole fraction is utilized to broaden the conduction band and create a three-dimensional electron sea (3DES) so that a coherent channel can be formed. Benefiting from the high electron density of the 3DES, this device exhibits an outstanding high-power performance. Deng et al. systematically compared the differences between MOCVD-SiNx and LPCVD-SiNx in terms of their Ohmic contact and related interfaces [16]. The growth interface of LPCVD-SiNx can suppress leakage effectively. Furthermore, it was discovered that LPCVD-SiNx devices can improve their RF output performance by creating a lower Ohmic contact resistance. Therefore, LPCVD-SiNx devices exhibit an excellent performance in small-sized modules working in low-voltage applications, which highlights their potential uses in small 5G terminals. Yang et al. report on the optimization of ultraviolet (UV) photodetectors with a TiO_2_ nanorod (NR)-containing active layer and a solid–liquid heterojunction (SLHJ) via the atomic layer deposition (ALD) of an Al_2_O_3_ passivation layer [17]. The oxygen vacancies in the TiO_2_ are effectively filled by the diffusing of Al, Ti, and O atoms between the Al_2_O_3_ passivation layer and TiO_2_ NRs during the annealing treatment. A difference of four orders of magnitude is observed in the photocurrent-to-dark-current ratio, demonstrating the advantages of an ALD-Al_2_O_3_ passivation layer in UV photodetectors.

Advanced modeling methods are vital to optimizing the design of devices. Recently, many researchers have become interested in artificial neural network (ANN) methods, using them in areas such as the subthreshold swing modeling of GaN HEMTs [18], GaN Ohmic contacts used for fabrication processes [19,20], and predicting the effect of statistical variability on Si junctionless nanowire transistors [21]. Within this area, Zhao et al. have proposed a compact ANN model generation methodology for GAA nanosheet FETs (NSFETs) used at advanced technology nodes [22]. The DC and AC characteristics of NSFETs can be reproduced by the optimized ANN model with a fitting error (MSE) of 0.01.

The characterization of traps is useful for improving device reliability, and the CV test, pulse IV, deep-level transient spectroscopy, simulation model, etc., are techniques frequently used to identify the state of traps in semiconductor devices [23,24,25,26,27]. Zou et al. reviewed the characterization techniques used for detecting bulk traps and interface traps in GaN HEMTs [28]. Electrical, optical, and junction capacitance methods have been widely used to probe the traps in GaN HEMTs. However, their low sensitivity, poor spatial resolution, and frequency range limit trap characterization, so further optimizations and innovations in their characterization techniques are needed.

Circuits and systems play a critical role in the explosive development of information technology, which has been regarded as a powerful engine in contemporary human civilization [29,30,31]. Interested in this topic, Jiang et al. proposed a lumped circuit based on a 3DIC physical structure and calculated the values of all the lumped elements in the circuit model and the transmission line model [32]. The 3DIC jitters, integrating DRAM logic and 3DIC designs into the simulation environment, were analyzed by the proposed CPSIA method, which determined that the timing uncertainty introduced by the 3DIC crosstalk ranged from 31 ps to 62 ps. Chen et al. proposed a novel frequency-domain broadband model (Sensi-Freq-Model) of the conduction susceptibility of integrated circuits, which accurately quantifies the conduction immunity of components in the frequency domain and builds a model of integrated circuits based on their quantized data [33]. The “Sensi-Freq-Model” can reduce the broadband modeling time by about 90% compared to the traditional ICIM-CI method, with a normalized mean square error (NMSE) of 18.5 dB. Li et al. designed a high-efficiency internally matched power amplifier with a 2.5 μm GaN HEMT [34]. An output power of 43.75 dBm, large-signal gain over 15.75 dBm, and PAE of 78.5% at 2.45 GHz were obtained from the proposed power amplifier, which is 13.4 × 13.5 mm^2^ in size. Xiao et al. presented a bandgap reference (BGR) source capable of operating over a wide input range [35]. The high-order curvature compensation method was used and a pre-regulation circuit was incorporated into the BGR. Then, a temperature coefficient (T_C_) of 0.88 ppm/°C and stable operation with variations in the power supply voltage were achieved over a temperature range of −40 °C to 130 °C.

In conclusion, this Special Issue showcases the dynamic landscape of semiconductor materials, devices, and systems, highlighting the innovations seen across a broad spectrum of research areas. From the exploration of wide- and ultrawide-bandgap materials to advancements in device optimization, modeling techniques, and characterization methods, these contributions underscore the field’s diversity and its pivotal role in technological progress. These studies not only push the boundaries of current semiconductor capabilities but also lay a strong foundation for future breakthroughs in energy efficiency, computational power, and miniaturization, driving us closer to the next era of electronic innovation.
